# Brain and Spinal Cord MRI Findings in Thai Multiple Sclerosis Patients

**DOI:** 10.3390/jimaging9020027

**Published:** 2023-01-26

**Authors:** Thippayaporn Lopaisankrit, Jureerat Thammaroj

**Affiliations:** Department of Radiology, Faculty of Medicine, Khon Kaen University, Khon Kaen 40000, Thailand

**Keywords:** multiple sclerosis, McDonald criteria (2017), MRI, Thai

## Abstract

Background: Previous studies have demonstrated different MRI characteristics in Asian and Western patients with multiple sclerosis (MS). However, the number of studies performed on Thai patients is still limited. Furthermore, these studies were conducted before the revision of the McDonald criteria in 2017. Methods: A retrospective descriptive study was performed on Thai patients diagnosed with MS, according to the McDonald criteria (2017), in a tertiary care hospital in Thailand. Results: Thirty-two patients were included (twenty-seven female and five male patients). The mean age was 37.8 years. Most (28 patients) had relapsing remitting MS. Brain MRIs were available for all 32 patients, all of which showed abnormalities. The most common locations were the periventricular regions (78.1%), juxtacortical regions (75%) and deep white matter (62.5%). Dawson’s fingers were identified in 20 patients (62.5%). Tumefactive MS was noted in two patients. Gadolinium-enhancing brain lesions were noted in nine patients (28.1%). Optic nerve lesions were found in seven patients. Six of the seven patients showed short segmental lesions with predominant posterior-half involvement. Spinal MRIs were available for 26 patients, with abnormalities detected in 23. Most (11 patients) had lesions both in the cervical and in the thoracic spinal cord. In total, 22 patients (95.7%) showed lesions at the periphery, most commonly at the lateral column. Fifteen patients showed lesions shorter than three vertebral segments (65.2%). Enhancing spinal lesions were noted in 14 patients. Dissemination in space was fulfilled in 31 patients (96.9%). Conclusion: Some of the MRI findings in our study were similar to those of previous studies in Thailand and Asia, emphasizing the difference between Asian and Western MS.

## 1. Introduction

Multiple sclerosis (MS) is the most common demyelinating disease in the central nervous system. It most often occurs in Caucasian female adults, aged 20–40 years. Patients may present with various neurological symptoms, depending on the location involved. After clinical onset, patients develop different clinical courses, most commonly relapsing and remitting [1].

Currently, the diagnosis of MS relies on the McDonald criteria (2017), which uses clinical history and physical examination and magnetic resonance imaging (MRI) combined with an oligoclonal bands in cerebrospinal fluid (CSF) for the evaluation of both the dissemination in space (DIS) and the dissemination in time (DIT) [2]. The use of MRI helps to differentiate other CNS diseases, evaluating treatment responses and following up disease progression [3].

Typical MRI findings in MS patients include round-shaped lesions exhibiting hyper signal intensity in T2-weighted (T2W) images, mostly in perivenular areas. Imaging characteristics that are sensitive for MS include lesions at callososeptal interfaces and lesions perpendicular to the lateral ventricles, in keeping with the axis of the medullary veins, or “Dawson’s fingers”. In T1-weighted (T1W) images, lesions may show hypo signal intensity; these are called T1 black-hole lesions. With gadolinium administration, active lesions may show enhancement in different patterns, including diffuse, nodular and ring-like enhancement. Incomplete ring enhancement may be seen, with the open ring located toward the gray matter [4,5]. According to the McDonald criteria (2017), DIS requires lesions involving at least two of the following four areas: the periventricular areas, cortical and/or juxtacortical areas, infratentorial areas (brainstem and cerebellum) and spinal cord [2]. The spinal cord lesions found in MS patients tend to be shorter than those found in neuromyelitis optica (NMO) patients; they are mostly located in the peripheral spinal cord (dorsal and lateral columns) and are found in the cervical spinal cord more often than in the thoracic spinal cord [4]. Optic nerve lesions are not included in the McDonald criteria (2017). However, previous research showed that the optic nerve lesions in MS patients tended to be unilateral short lesions and involve the anterior half of the optic nerves (ON) rather than the posterior half [6].

In addition to the typical findings mentioned, there are few atypical imaging findings in MS. Tumefactive MS may manifest as a relatively well-defined mass-like lesion of more than 2 centimeters, mimicking a brain tumor [7]. Baló’s concentric sclerosis (BCS) manifests as a target-like lesion made up of alternating concentric rings of demyelination and relatively preserved myelin [8].

Previous studies showed different MRI findings between Asian and Western patients with MS in which the etiology is still unknown. Previous studies categorized MS phenotypes in Asian populations as classic (conventional, or Western) MS and optic–spinal MS. The proportions of these subtypes vary among studies. Optic–spinal MS occurred with older patients (>35 years), with fewer brain lesions and more optic nerve and spinal involvement. When spinal involvement occurs, the lesion is typically longer [9]. Disability progresses more rapidly with more frequent relapses. A CSF analysis of optic–spinal MS showed higher white blood cell counts and protein levels and fewer cases showed positive CSF oligoclonal bands [10]. 

Kira performed a study on Japanese MS patients (2003) [9]. He proposed that the fewer brain lesions and longer spinal cord lesions in optic–spinal MS are linked with different human leukocyte antigens and immune-mediated mechanisms. 

Chong et al. performed a multicentered study in seven regions throughout Asia. About 50% of the patients had optic–spinal MS and found that the mean length of the spinal cord lesions in all the patients was 3.6 ± 3.3 vertebral bodies, with 2.5 ± 3.3 vertebral bodies in the classical MS subgroup and 4.2 ± 3.6 vertebral bodies in the optic–spinal MS subgroup (*p*-value of 0.001) (2006) [11]. 

Li et al. [10] also performed a study on Chinese MS patients, of whom 35.9% had optic–spinal MS. The longitudinal fusion of the spinal lesions of at least three vertebral segments was found in 94.1% of the patients with optic–spinal MS and only 48.2% of the patients with classic MS (*p*-value = 0.002). Spinal cord atrophy was found more commonly in optic–spinal MS. They also found that the brain MRIs in classic MS showed concentrated lesions along the periventricular areas, extending to the deep white matter. When in optic–spinal form, MS lesions are usually confined to the periventricular areas and brainstem (2014) [10].

In Thailand, a study performed by Tritrakarn et al. (2018) that compared the different MRI findings of patients with MS and NMO showed findings that were suggestive of MS, including signs of Dawson’s finger, as well as periventricular inferior temporal lobe and corticospinal tract lesions [12]. Of the few studies available in Thailand, only the study performed by Jitpratoom et al. (2012) [13] calculated the mean spinal cord lesion length. The authors found a mean spinal cord lesion of about 1.29 vertebral segments, which was no different from that seen in Western MS patients [13]. The studies performed with Thai MS patients are still limited and were performed prior to the revision of the McDonald criteria in 2017 [14]. The objective of this research was to evaluate the brain and spinal cord MRI findings in Thai patients who were diagnosed with MS, according to the McDonald criteria (2017).

## 2. Methods

We retrospectively searched papers and electronic medical records of patients in Srinagarind hospital, affiliated to the Faculty of Medicine of Khon Kaen University, a tertiary care hospital in northeast Thailand.

All Thai patients aged over 18 years, who were diagnosed with MS between 1st January 2007 and 30th September 2021 according to McDonald criteria (2017), with at least brain or spinal MRI images available in picture-archiving and communication system (PACS) at our hospital were recruited. Patients aged lower than 18 years; patients of races other than Thai; patients with final diagnosis of other demyelinating diseases, such as NMO, anti-myelin oligodendrocyte glycoprotein (anti-MOG) and acute disseminated encephalomyelitis (ADEM); patients whose MRI images were not available at our PACS; patients with suboptimal MRI imaging quality for interpretation; and patients without adequate clinical information to support their diagnosis were excluded. 

A total number of 32 patients met these inclusion and exclusion criteria. These patients had a confirmed diagnosis of MS by neurology staff at our institute. Demographic data, clinical information, laboratory results and MRI findings, recorded using Research Electronic Data Capture (REDCap) system hosted by Khon Kaen University, were recorded and analyzed using Microsoft Excel.

Basic demographic data, including age, age at clinical onset, sex, presenting symptoms and clinical subtypes (classified as relapsing–remitting MS, primary progressive MS, secondary progressive MS and progressive relapsing MS) were recorded. Laboratory results of CSF and serum oligoclonal band were recorded as positive or negative if available.

All brain or spinal MRIs of the patients performed between 1st January 2007 and 30th September 2021, regardless of whether they were performed at an early time after onset of the clinical or during follow-up periods, were reviewed by a neuroradiologist with more than 20 years’ experience. If the brain/spinal MRIs of the patients were performed more than once, the study with largest number of lesions in each category (brain MRI or spinal MRI) was selected. Our analysis of both the brain and spinal MRIs included the location, characteristics, number of lesion(s) and maximal diameter/length of the largest lesion in T2-weighted sequence. Presence of gadolinium-enhancing lesion(s) was also recorded. Visual assessment of the brain/spinal cord atrophy was also conducted. Finally, we determined whether the patients’ brain and spinal MRIs met McDonald criteria (2017) for dissemination in space. The MRI scanners available at our institute include a 1.5 Tesla scanner (ManetomArea, Siemens Medical Solution, Erlandgen, Germany) and a 3.0 Tesla scanner (Philips Achieva, Philips Medical System, Netherland, B.V.)

The data collected were analyzed by STATA10 program using descriptive statistics. Qualitative data, such as sex, presenting symptoms, disease subtypes, lesion location, lesion characteristic and presence of gadolinium-enhancing lesion were demonstrated as frequency and percentage. Maximal diameter/length of the lesions and number of lesions were categorized into frequency tables by determining the range of the data set (the maximum minus the minimum value) and the number of classes to create class width, after which they were presented as frequency and percentage. Continuous numbers, including age, lesion number and lesion size and length were tested for normal data distribution using Shapiro–Wilk W test. Data with normal distribution were presented using mean and standard deviation (SD), while data without normal distribution were presented using median and interquartile range (IQR).

This study was approved by the Ethics Committee for Human Research, Khon Kaen University. The patients’ personal information was concealed. Data were collected under newly assigned identification number and general interpretation was conducted.

## 3. Results

Thirty-two Thai patients with a diagnosis of MS according to the McDonald criteria (2017) were recruited. Of these, twenty-seven were female (84.4%) and only five were male (15.6%). The median age of these patients was 36 years (ranging from 20 to 65 years), while the median age at clinical onset was 27 years. Two most common clinical presentations were cerebral syndrome (17 patients, 53.1%) and myelitis (15 patients, 46.9%). Optic neuritis was the third most common presentation, found in nine patients (28.1%). The disease subtypes among these patients were mostly relapsing–remitting (28 patients, 87.5%), while only two patients (6.25%) with each of primary progressive and secondary progressive clinical courses were found. The CSF oligoclonal band was available in 16 (50%) of the recruited patients. Positive CSF oligoclonal bands with negative serum oligoclonal bands were found in eight patients (50% of all patients with available oligoclonal band results; Table 1).

**Figure 1:**
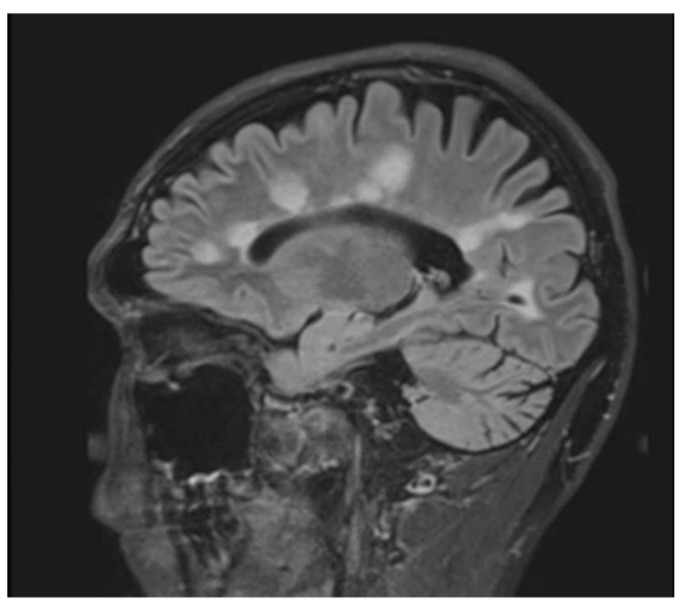
Sagittal fluid attenuated inversion recovery (FLAIR) image shows lesions perpendicular to lateral ventricle giving classic finger-like appearance, Dawson’s fingers.

**Figure 2:**
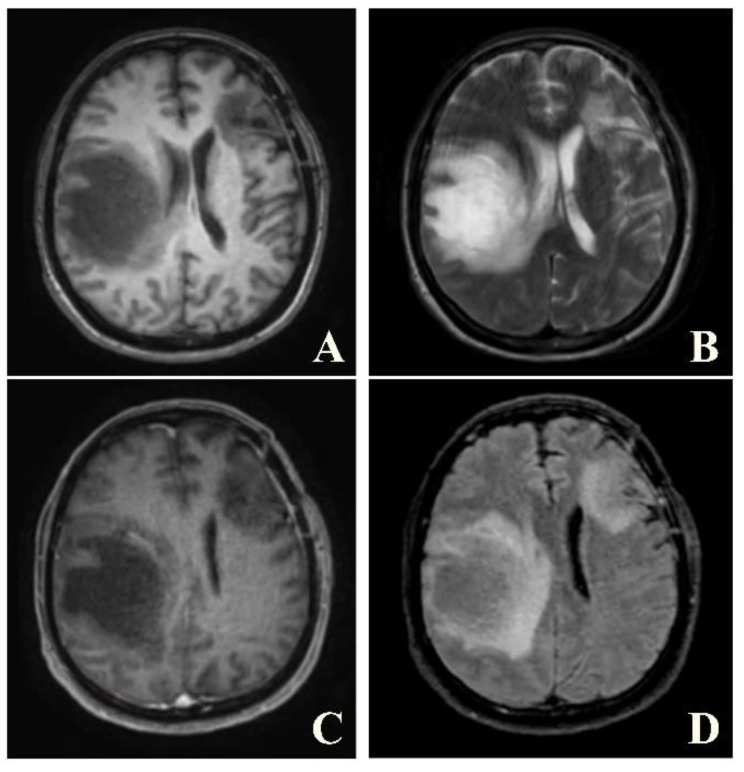
Axial T1W (**A**), T2W (**B**), post-contrast T1W (**C**) and post-contrast FLAIR (**D**) images of the same patient reveal tumor-like lesion with surrounding vasogenic edema at left frontal and right parietal lobes. The patient was suspected to have high-grade glioma and underwent biopsy. Pathological result was consisted with demyelination, yielding a final diagnosis of tumefactive MS. The patient was given treatment with high-dose steroids.

**Figure 3:**
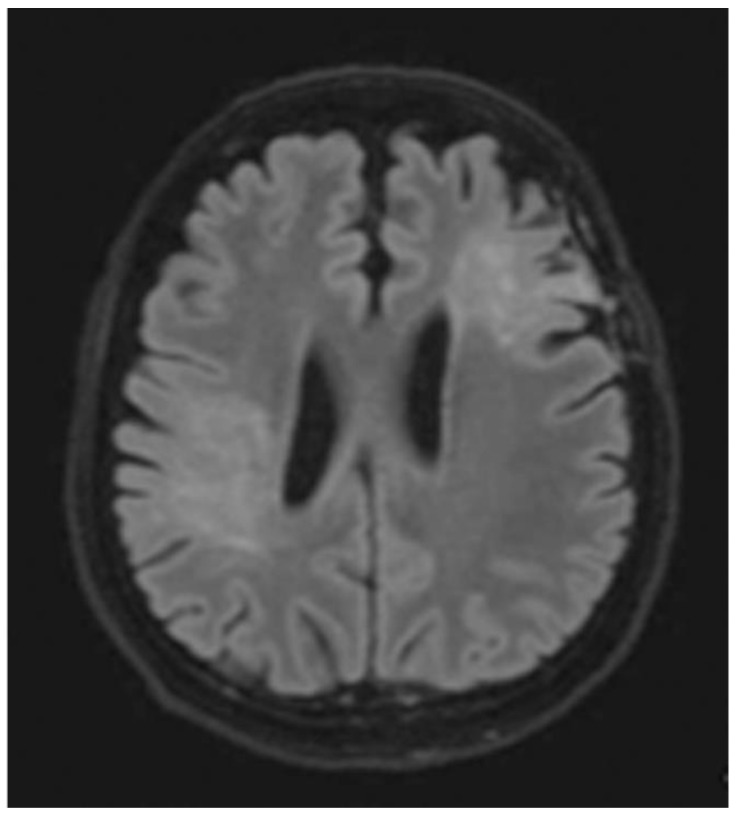
A follow-up axial post-contrast FLAIR image of the same patient performed one year after the diagnosis, showing marked improvement in lesions.

**Figure 4:**
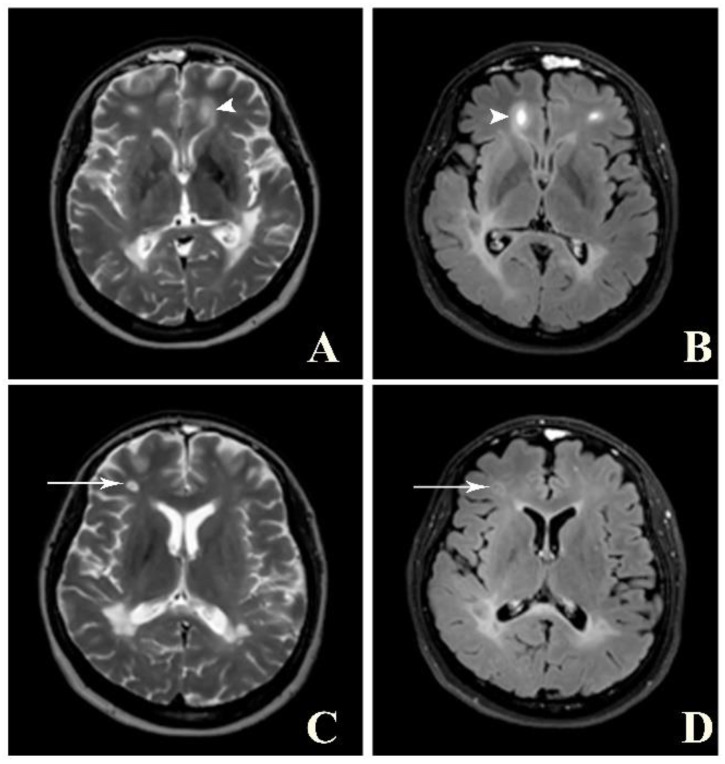
Balo’s concentric sclerosis. **A** and **B**: Axial T2W (**A**) and post-contrast FLAIR (**B**) images show a few lesions with alternating band of high and low signal intensity at bilateral frontal lobes (arrowhead). **C** and **D**: Axial T2W (**C**) and post-contrast FLAIR (**D**) images of the same patient show faint central and peripheral enhancement at the right frontal lobe lesion (arrow).

**Figure 5:**
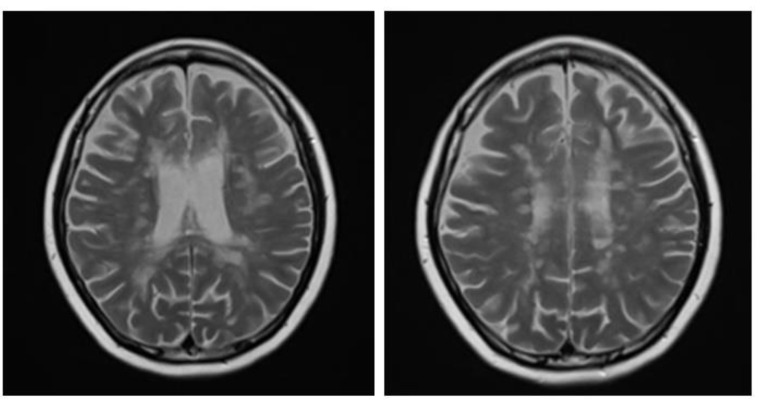
Axial T2W images of the same patient showing extensive hypersignal–intensity lesions scattered at juxtacortical region, subcortical region, deep white matter and periventricular white matter of bilateral cerebral hemispheres.

A brain MRI was available for all 32 patients, all of whom showed abnormalities. Most of the patients (19, 59.4%) were subjected to MRI soon after clinical presentation and during follow-up. The lesions were most commonly identified in the periventricular regions (78.1%), juxtacortical regions (75%) and deep white matter (62.5%). Cortical lesions were identified in only eight patients (25%). Other locations included in the McDonald criteria (2017) were the brainstem and cerebellum, which were found in 59.4 and 21.9% of all patients, respectively. The most common characteristic of the lesions was a round/oval discrete appearance (29 patients, 90.6%). A T1W-blackhole and classic Dawson’s finger (Figure 1) were equally identified in 20 patients (62.5%). A tumefactive form was found in two patients (Figure 2 and Figure 3). There were also two patients with target-like lesions, resembling Balo’s concentric sclerosis (Figure 4). The maximal diameter of the lesions was most commonly less than 13 mm (34.4%). A few patients showed lesions measuring more than 52 mm (9.4%), particularly those with confluent lesions. The total number of lesions in the T2W images was most commonly counted at less than 23 (50.0%), while there were up to four patients with extensive lesions, or more than 47 (12.5%) (Figure 5). Gadolinium-enhancing lesions were identified in nine patients (28.1%), who mostly only had one or two of these lesions (in five out of nine patients). Of these, the most common enhancing pattern was nodular enhancement (in four patients). The classic incomplete ring enhancement was identified in two patients, while complete ring enhancement was found in one patient. A visual assessment of the brain volume found brain atrophy in four patients (12.5%). Two patients with tumefactive MS showed brain swelling (Table 2).

**Figure 6:**
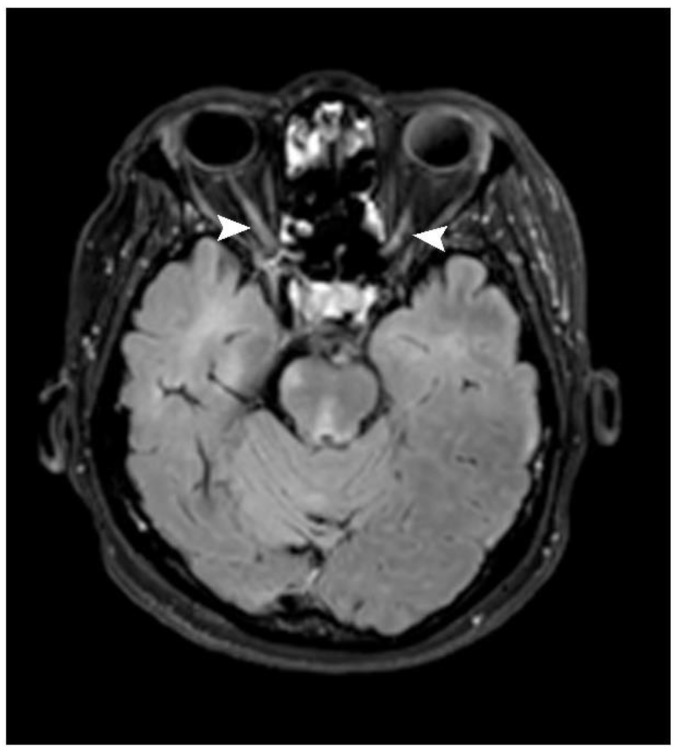
Axial post-contrast FLAIR image shows patchy enhancement at posterior halves of bilateral optic nerves (arrowhead) in keeping with bilateral optic neuritis.

Optic nerve lesions were identified in seven patients (21.9%). A unilateral lesion and bilateral lesions were found in three and four patients (42.9 and 57.1%), respectively. A long-segment lesion (longer than 50% of the optic nerve length) was identified in only one patient, while the remainder showed short-segment lesions (six patients, 85.7%). Of these, four showed posterior-half lesions (66.7%; Figure 6, Table 2).

**Figure 7:**
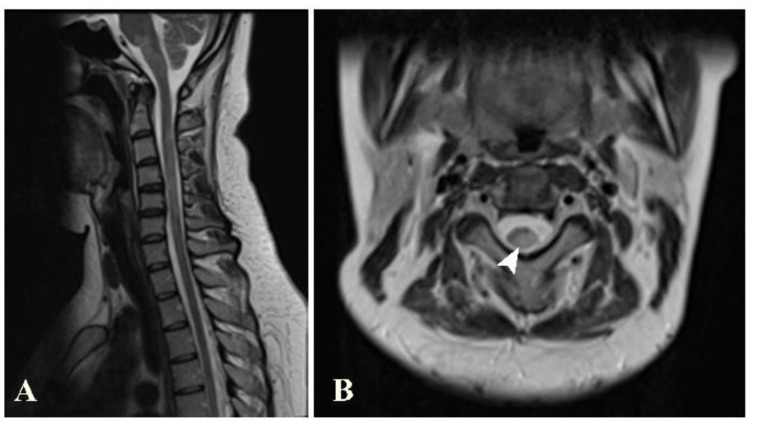
Sagittal T2W image (**A**) of the cervical spine shows brainstem and several spinal cord lesions. Axial T2W image (**B**) shows lesions involving both central and peripheral regions of the spinal cord (arrowhead), mainly in lateral and posterior columns.

A spinal MRI was available for 26 of the patients. Of these, 23 (88.5%) showed an abnormal spinal MRI. These spinal MRIs were most frequently performed during the follow-up period (in 10 out of 26 patients, 38.5%). Almost half of the patients with abnormal MRIs showed lesions in both the cervical and the thoracic spinal cord (11 patients, 47.8%). Seven and four patients showed lesions at the cervical spinal cord only and the thoracic spinal cord only, respectively. The locations of these lesions in the axial views were most common by far in the peripheral areas (22 patients, 95.7%). Only five and two patients had lesions in the central spinal cord and whole-cross-sectional cord involvement, respectively. Regarding the spinal cord column involvement in the peripheral lesions, the most commonly involved column was the lateral column (19 patients, 82.6%), followed by the posterior and the anterior column (ten and seven patients, respectively) (Figure 7). The lesions were mainly round/oval (91.3%). The maximal length of the longest lesion, classified into shorter than three and at least three vertebral segments, showed that the predominant lesion length was shorter than three vertebral segments (15 patients, 65.2%). The median and mean lesion length were about 2 and 2.2 vertebral segments, respectively. Most of the patients showed only one or two spinal lesions (16 patients, 69.6%), with only two of the patients developing more than five lesions (8.7%). The presence of spinal gadolinium-enhancing lesions was noted in 14 patients (60.9%), with one or two enhancing lesion(s) found in each. The most common enhancing pattern was nodular enhancement (39.1%), while incomplete ring enhancement was also noted in the spinal cord in two patients (8.7%). A visual assessment found spinal-cord atrophy in four patients (12.5%; Table 3).

**Figure 8:**
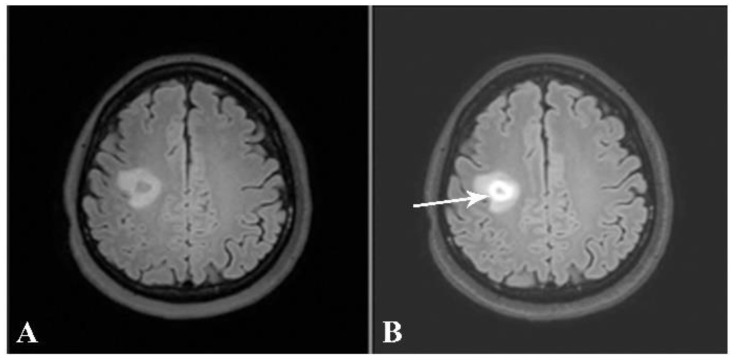
Axial precontrast FLAIR (**A**) and postcontrast FLAIR (**B**) images show complete ring-enhancing lesion (arrow) with minimal surrounding vasogenic edema. No significant pressure effect is shown. A presumptive diagnosis of small tumefactive MS was made, although there were no other lesions, either in the brain or in the spinal cord. The patient was given high-dose intravenous steroid.

**Figure 9:**
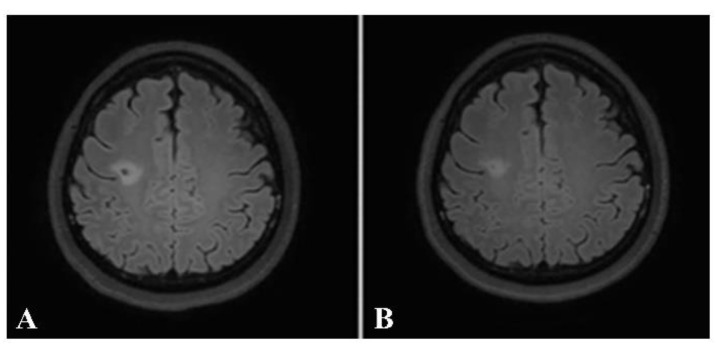
Follow-up axial postcontrast FLAIR images of the same patient after treatment with steroids at 2 and 6 months (**A** and **B**, respectively), showing decreased size of the lesion without residual enhancement.

Taking into consideration each patient’s brain MRI and spinal MRI, if available, dissemination in space (DIS), according to the McDonald criteria, was found in 31 out of the 32 patients (96.9%). The only patient whose MRIs did not demonstrate DIS was the one with a brain MRI typical of tumefactive MS. The patient received a high dose of intravenous methylprednisolone. After follow-up, the lesion decreased in size and no new lesions were detected (Figure 8 and Figure 9).

## 4. Discussion

This study showed some similarities with previous studies conducted in Thailand [13]. Firstly, similar age groups and a high female-to-male ratio were noted in both studies. Regarding the MRIs, we also found that the periventricular region was the most common lesion location (78.1% in our study and 52.8% in Jitpratoom’s study). Jitpratoom et al. [13] also found that the juxtacortical region was the second most common location. Regarding the spinal MRI, Jitpratoom found that the mean length of the spinal cord lesions was 1.29 vertebral segments, whereas our study showed a mean spinal cord lesion length of about 2.2 vertebral segments [13]. Another study performed in Thailand [12] showed negative spinal MRIs in 41.9% of all the patients, with the remaining patients with positive MRIs showing cervical and thoracic spinal cord lesion in 48.4 and 29.0%, respectively. Our study showed a much higher proportion of positive MRIs (23 out of 26 patients with available spinal MRIs, 88.5%). The performance of spinal MRIs on patients with suspected spinal lesions only in our institute may have contributed to the high positive rate. Our study also identified that most of the patients had both cervical and thoracic spinal cord lesions (11 patients, 47.8%), with a cervical lesion alone or a thoracic lesion alone in seven and four patients, respectively. Tritrakarn et al. also collected data about optic nerves, showing optic nerve lesions in four of thirty patients, of whom three patients had lesions in the anterior half of the optic nerve [12]. Our study showed optic nerve lesions in seven of thirty-two patients. Of these, six short-segmental lesions were identified, of which four were located in the posterior half of the optic nerve.

In a previous study in Asia, Chong et al. (2006) found a mean length of spinal cord lesions of up to 3.6 ± 3.3 vertebral bodies [11], which was longer than that in our study. However, our study still showed longer spinal cord lesions compared with a study performed in a Western country [15], which showed predominant multiple short spinal cord lesions (with a median number of three lesions and a median length of 0.8 vertebral segments). Li et al. (2014) found that in the early onset of classical MS, spinal cord swelling may be shown, while in optic–spinal MS, spinal cord atrophy may be seen [10]. In our study, two patients with cord swelling and four patients with cord atrophy were found. A previous Western study observed Dawson’s fingers in 31–37 out of 40 patients (78–93%), depending on the observers [16]. A previous study, in China, showed Dawson’s finger in 59 of 80 patients (73.8%) [17]. In addition to the predominance of periventricular lesions, as mentioned above, our study also showed periventricular perpendicular lesions, or Dawson’s fingers, in as many as 20 patients (62.5%).

Regarding the revision of the McDonald criteria in 2017, cortical lesions were added (only juxtacortical lesions were taken into consideration in the McDonald criteria from 2010). Our study found eight patients (25%) with cortical lesions. However, all of these eight patients also had a juxtacortical lesion. This resulted in a questionable increment of the diagnostic sensitivity. The use of CSF oligoclonal bands in the diagnosis of dissemination in time (DIT) was also added. Positive CSF oligoclonal bands were noted in 50% of our patients. These findings emphasized that the revision of the McDonald criteria (2017) may increase the sensitivity in the diagnosis of MS. In addition to positive CSF oligoclonal bands, the presence of gadolinium-enhancing lesions can also be considered to demonstrate DIT. Our study showed the presence of a gadolinium-enhancing lesions within the brain in the spines of at least nine patients (28.1%) and up to fourteen (60.9%).

Our study also showed DIS in up to 31 of the 32 patients (96.9%), which was higher than in the previous studies in Thailand, which used prior versions of the diagnostic criteria.

There were a few limitations in our study. Firstly, the number of patients included may have been slightly small. As our institute is a tertiary-care hospital, many patients with a confirmed diagnosis of MS may have received their MRI at their primary-care center prior to their diagnosis, resulting in unavailable MRI images in the PACS at our hospital. Secondly, the study period included the transition from paper medical records to electronic databases. Some patient medical records were lost. Patients with insufficient data supporting the confirmation of their diagnosis of MS were excluded from our studies. An example of this would be if a patient’s medical records were available for only one clinical event. Thirdly, CSF oligoclonal bands are not available at our hospital. Only half of our patients had an oligoclonal band, resulting in limitations in the interpretation of the positive rate. Moreover, the studies included patients receiving different treatments; some only received steroids, while others received disease-modifying agents. We analyzed the MRI scans with the largest number of lesions, which we considered to be the scans that were most likely to represent the times when the patients had clinical attacks. Lastly, our study did not distinguish between patients with classical MS and those with optic–spinal MS due to the small number of patients included, which limits the scope of comparison with the previous Asian studies, since the proportion of patients with the optic–spinal form may have been different. A further multi-centered study in Thailand with a larger number of subjects is recommended.

In conclusion, some of the MRI findings in our study showed similar results to the previous studies performed on Thai and Asian patients, emphasizing the differences between the MRI findings from Asian and Western MS patients. 

## Figures and Tables

**Table 1:** Demographic data, clinical presentation, and laboratory results.

	Frequency	Percentage
	(N = 32 Patients)	
Sex		
Female	27	84.4%
Male	5	15.6%
Age (years, median)	36 (range 20–65 years)	
Age at clinical onset (years, median)	27 (range 13–58 years)	
Clinical presentation		
Cerebral syndrome	17	53.1%
Myelitis	15	46.9%
Optic neuritis	9	28.1%
Brainstem syndrome	2	6.3%
Cerebellar syndrome	1	3.1%
Disease course subtype		
Relapsing remitting MS	28	87.5%
Primary progressive MS	2	6.25%
Secondary progressive MS	2	6.25%
Progressive relapsing MS	0	0%
CSF oligoclonal band		
Not available	16	50%
Available	16	50%
Positive	8	50% of all available results
Negative	8	50% of all available results

**Table 2:** Brain MRI findings (brain MRIs were available for all 32 patients).

	Frequency	Percentage
	(N = 32 Patients)	
Unavailable brain MRI	0	
Available MRI	32	
Normal brain MRI	0	0%
Abnormal brain MRI	32	100%
Timing of applicable brain MRI		
Early after clinical presentation	6	18.8%
During follow-up	7	21.9%
Both	19	59.4%
Location of lesion(s)		
Periventricular	25	78.1%
Juxtacortical	24	75%
Cortical	8	25%
Brainstem	19	59.4%
Cerebellum	7	21.9%
Deep white matter	20	62.5%
Deep gray nuclei	14	43.8%
Optic nerve(s)	7	21.9%
Unilateral ON	3	42.9% (Of 7 patients)
Bilateral ONs	4	57.1% (Of 7 patients)
Long segment (>50% ON length)	1	14.3% (Of 7 patients)
Short segment (<50% ON length)	6	85.7% (Of 7 patients)
Anterior half of ON	2	33.3% (Of 7 patients)
Posterior half of ON	4	66.7% (Of 7 patients)
Characteristics of lesion(s)		
Round/oval shaped lesion in T2W	29	0.906
Nodular/punctate lesion in T2W	7	0.219
Confluent high T2W signal lesions	8	0.25
T1W black hole	20	0.625
Dawson’s finger	20	0.625
Atypical pattern		
Tumefactive lesion	2	0.063
Target-like	2	0.063
Maximal diameter of the largest lesion	median (min–max)	median (IQR)
median (range)	18.8 (2.6–63.7)	18.8 (11.5–32.35)
Maximal diameter of the largest lesion		
1–13 mm	11	34.4%
13.1–26 mm	8	25.0%
26.1–39 mm	8	25.0%
39.1–52 mm	2	6.3%
>52 mm	3	9.4%
Presence of gadolinium-enhancing lesion		
Not present	23	71.9%
Present	9	28.1%
Enhancing pattern		(Of 9 patients with enhancing lesion)
Nodular enhancement	4	44.4%
Diffuse enhancement	3	33.3%
Incomplete ring enhancement	2	22.2%
Complete ring enhancement	1	11.1%
Total number of lesion(s) in T2W	median (min–max)	median (IQR)
median (range)	22 (1–115)	22 (4–37)
Total number of lesion(s) in T2W		
1–23 lesion(s)	16	50.0%
24–46 lesions	12	37.5%
47–69 lesions	1	3.1%
70–92 lesions	1	3.1%
>92 lesions	2	6.3%
Total number of gadolinium-enhancing lesion(s)	median (min–max)	median (IQR)
median (range)	0 (0–11)	0 (0–1)
Total number of gadolinium-enhancing lesion(s)		
None	23	71.9%
1–2 lesion(s)	5	15.6%
3–5 lesions	3	9.4%
>5 lesions	1	3.1%
Visual assessment of brain volume		
Normal according to his/her age	26	81.3%
Atrophy	4	12.5%
Swelling	2 (in patients with tumefactive lesion)	6.3%

**Table 3:** Spinal MRI findings (Spinal MRI was applicable in 26 patients).

	Frequency	Percentage
Unavailable spinal MRI	6	
Available spinal MRI	26	
Normal spinal MRI	3	11.5%
Abnormal spinal MRI	23	88.5%
Timing of applicable spinal MRI		(Of 26 patients with available MRI)
Early after clinical presentation	8	30.8%
During follow-up	10	38.5%
Both	8	30.8%
Level of lesion(s)		(Of 23 patients with abnormal MRI)
Cervical	7	30.4%
Thoracic	4	17.4%
Both cervical and thoracic	11	47.8%
Cervical + thoracic + lumbar	1	4.3%
Axial location of lesion(s)		(Of 23 patients with abnormal MRI)
Central	5	21.7%
Peripheral	22	95.7%
Lateral column	19	82.6%
Posterior column	10	43.5%
Anterior column	7	30.4%
Whole cross-section	2	8.7%
Characteristics of lesion(s)		(Of 23 patients with abnormal MRI)
Round/oval-shaped lesion in T2W	21	91.3%
Nodular/punctate lesion in T2W	4	17.4%
Confluent high-T2W-signal lesions	4	17.4%
T1W black hole	1	4.4%
Maximal length of the longest lesion (mm)		median (min–max); 25.6 (5.2–101.3)
median (range)		median (IQR); 25.6 (12–47.9)
Maximal length of the longest lesion (mm)		
5–24 mm	10	43.5%
24.1–44 mm	6	26.1%
44.1–64 mm	3	13.0%
64.1–84 mm	3	13.0%
>84 mm	1	4.4%
Maximal length of the longest lesion (compared with vertebral segments)		(Of 23 patients with abnormal MRI)
		median (min–max); 2 (1–5)
		median (IQR); 2 (1–3)
Maximal length of the longest lesion (compared with vertebral segments)		(Of 23 patients with abnormal MRI)
<3 segments	15	65.2%
≥3 segments	8	34.8%
Presence of gadolinium-enhancing lesion		(Of 23 patients with abnormal MRI)
Not present	9	39.1%
Present	14	60.9%
Enhancing pattern		(Of 14 patients with enhancing lesion)
Nodular enhancement	9	39.1%
Diffuse enhancement	5	21.7%
Incomplete ring enhancement	2	8.7%
Total number of lesion(s) in T2W		(Of 23 patients with abnormal MRI)
1–2 lesion(s)	16	69.6%
3–4 lesions	5	21.7%
>5 lesions	2	8.7%
Total number of gadolinium-enhancing lesion(s)		(Of 23 patients with abnormal MRI)
None	9	39.1%
1 lesion	10	43.5%
2 lesions	4	17.4%
Visual assessment of spinal cord volume		(Of 26 patients with available MRI)
Normal according to his/her age	26	81.3%
Atrophy	4	12.5%
Swelling	2	6.3%

## Data Availability

The data presented in this study are available on request from the corresponding author. The data are not publicly available due to the privacy of research participants.
